# Influence of andrographolide on the pharmacokinetics of warfarin in rats

**DOI:** 10.1080/13880209.2018.1478431

**Published:** 2018-07-08

**Authors:** Xiaoli Zhang, Xiaosu Zhang, Xiaocui Wang, Meijun Zhao

**Affiliations:** a Department of Nephrology, Yidu Central Hospital of Weifang, Shandong, China;; b Department of Nursing, Yidu Central Hospital of Weifang, Shandong, China

**Keywords:** Herb-drug interaction, metabolism, LC-MS/MS

## Abstract

**Context**: Andrographolide and *w*arfarin are often used together in clinics in China. However, the herb-drug interaction between andrographolide and warfarin is still unknown.

**Objective:** This study investigates the herb-drug interaction between andrographolide and warfarin *in vivo* and *in vitro*.

**Materials and methods:** A sensitive and reliable LC-MS/MS method was developed for the determination of warfarin in male Sprague-Dawley rats plasma, and then the pharmacokinetics of orally administered warfarin (0.5 mg/kg) with or without andrographolide (30 mg/kg/day for 7 days) pretreatment was investigated. In addition, Sprague-Dawley rat liver microsomes incubation systems were used to support the *in vivo* pharmacokinetic data and investigate its potential mechanism.

**Results:** The method validation results showed that a sensitive and reliable LC-MS/MS method was developed for the determination of warfarin in rat plasma samples. The pharmacokinetic results indicated that co-administration of andrographolide could increase the systemic exposure of warfarin significantly, including area under the curve (118.92 ± 18.08 *vs.* 60.58 ± 9.46 μg × h/mL), maximum plasma concentration (3.32 ± 0.41 *vs.* 2.35 ± 0.25 μg/mL) and *t*_1/2_ (22.73 ± 3.28 *vs.* 14.27 ± 2.67 h). Additionally, the metabolic stability of warfarin increased from 23.5 ± 4.7 to 38.7 ± 6.1 min with the pretreatment of andrographolide, and the difference was significant (*p* < 0.05).

**Discussion and conclusion:** In conclusion, andrographolide could increase the systemic exposure of warfarin in rats when andrographolide and warfarin were co-administered, and possibly by slowing down the metabolism of warfarin in rat liver by inhibiting the activity of CYP3A4 or CYP2C9.

## Introduction

Warfarin is recommended most widely for the prevention of systemic embolism, venous thromboembolism, and stroke associated with atrial fibrillation (Friberg and Oldgren 2017; Liu et al. 2018; Norby et al. 2017; Patel et al. 2017). The annual prescriptions of warfarin typically occur in 0.5–1.5% of the population (Winkelmayer et al. 2012). Although novel oral anticoagulants (NOACs), such as apixaban, dabigatran, edoxaban and rivaroxaban, do not require more special laboratory monitoring or dose adjustment like warfarin does, warfarin has remained in use for over 60 years and is still the most common oral anticoagulant drug (Pirmohamed et al. 2015; Bala et al. 2017; Nagata et al. 2017). This is mainly because warfarin can exert anticoagulant effects for several days after the subject stops taking it, while the NOACs do not have this merit.

In various countries and regions, especially in China, physicians prefer to combine herbal medicine with warfarin to achieve better effects and to prevent or reduce the side effects (Chua et al. 2015; Li et al. 2016; Choi et al. 2017; Di Minno et al. 2017; Liu et al. 2018). Certain herbs, including aloe, jalap, cascara and rhubarb, have been found to affect the metabolism of warfarin via cytochrome P450s (CYP) and UDP-glucuronosyltransferases pathway (Greenblatt and von Moltke 2005; Lee and Fermo 2006; Milic et al. 2014). Therefore, it is important to study the interactions between herbal medicines and warfarin so as to determine their optimal clinical applications.

Andrographolide is one of principal constituents of traditional herbal medicine *Andrographis paniculata* (Burm) Nees, which is one of the most important medicinal plants used in China, Thailand and Ayurvedic medicine to treat gastric disorder, colds, influenza, and other infectious diseases (Lim et al. 2012; Aromdee 2014; Chua 2014; Gupta et al. 2017). It has been reported that andrographolide has many bioactivities, such as anti-inflammatory, antiplatelet aggregation, antihyperglycemic, choleretic, anti-tumor and anti-HIV activities (Xie et al. 2016; Yuan et al. 2016; Yang R et al. 2017; Zhao et al. 2017). In China, preparations containing andrographolide as over-the-counter (OTC) drugs can be easily obtained from drug stores, and are often used in combination with antibiotics or other anti-infectious agents (Wu et al. 2008; Tsai et al. 2013; Chen et al. 2016). As a supplement, andrographolide is also used widely around the world. Andrographolide and warfarin are often used together in clinics in China, however, the herb-drug interaction between andrographolide and warfarin is still unknown.

Therefore, this study investigates the effects of andrographolide on the oral pharmacokinetics of warfarin in rats and explores the possible mechanisms of herb-drug interaction using rat liver microsomes incubation systems.

## Materials and methods

### Chemicals and reagents

Warfarin (purity >98%) and quercetin (internal standard, purity >98%) were purchased from the National Institute for the Control of Pharmaceutical and Biological Products (Beijing, China). β-Nicotinamide adenine dinucleotide phosphate (NADP) and lucifer yellow were provided by Sigma (St. Louis, MO, USA). Rat liver microsomes were purchased from BD Gentest (Woburn, MA, USA). Acetonitrile and methanol were purchased from Fisher Scientific (Fair Lawn, NJ, USA). Formic acid was purchased from Anaqua Chemicals Supply Inc. Limited (Houston, TX, USA). Ultrapure water was prepared with a Milli-Q water purification system (Billerica, MA, USA). All other chemicals were of analytical grade or better.

### Animal experiments

Male Sprague-Dawley (SD) rats weighing 220–250 g were provided by the Experimental Animal Center of Weifang Medical University (Weifang, China). The animal care, use and experimental protocols were also approved by the animal care committee of Weifang Medical University. Rats were kept at standard laboratory condition (at 25 °C with 60 ± 5% humidity and a 12 h dark-light cycle). Tap water and normal chow were given *ad libitum*. All of the experimental animals were kept under the above conditions for a three-day acclimation period and fasted overnight before the experiments.

### LC-MS/MS determination of warfarin

The determination of warfarin was performed using an Agilent 1290 series liquid chromatography system and an Agilent 6470 triple-quadruple mass spectrometer (Palo Alto, CA, USA). The chromatographic analysis of warfarin was performed on a Waters X-bridge C18 column (3.0 × 100 mm, i.d.; 3.5 μm) at room temperature. The mobile phase was water (containing 0.1% formic acid) and acetonitrile (35:65, v:v) at a flow rate of 0.3 mL/min. The mass scan mode was negative MRM mode. The precursor ion and product ion are *m/z* 307.0 → 250.1 for warfarin, and m/z 301.0 → 121.1 for quercetin (internal standard), respectively. The collision energy for warfarin and quercetin were 20 and 25 ev, respectively. The MS/MS conditions were optimized as follows: fragmentor, 110 V; capillary voltage, 3.5 kV; nozzle voltage, 500 V; nebulizer gas pressure (N_2_), 30 psig; drying gas flow (N_2_), 10 L/min; gas temperature, 350 °C; sheath gas temperature, 400 °C; and sheath gas flow, 11 L/min.

### Plasma sample preparation

Each plasma sample (100 μL) was spiked with 20 μL methanol. The mixture was then extracted with 180 μL of the internal standard methanol solution (500 ng/mL) by vortexing for 1 min. After centrifugation at 15,000 rpm for 10 min, the supernatants were transferred into an injection vial and a 3 μL aliquot was injected into the LC-MS/MS system for the analysis.

### Preparation of standard and quality control samples

A stock solution of warfarin was prepared in acetonitrile at a concentration of 2 mg/mL. The stock solution of the quercetin was prepared in acetonitrile at a concentration of 1 mg/mL. Calibration standard samples for warfarin were prepared in blank rat plasma at concentrations of 10, 20, 50, 100, 200, 500, 1000, 2000 and 5000 ng/mL. The quality control (QC) samples were prepared at low (20 ng/mL), medium (200 ng/mL), and high (4000 ng/mL) concentrations in the same way as the plasma samples for calibration, and QC samples were stored at −40 °C until analysis.

### Method validation

The LC-MS/MS method validation was performed in accordance with the United States Food and Drug Administration (FDA) guidelines (2013).

### Selectivity

The selectivity of the method was investigated by comparing the chromatograms from six different batches of blank rat plasma with the corresponding spiked plasma to exclude interference from endogenous substances and metabolites.

### Linearity and sensitivity

The calibration curve was obtained using nine concentrations (10, 20, 50, 100, 200, 500, 1000, 2000 and 5000 ng/mL) which were processed and determined as described above. The calibration curves for warfarin were constructed by plotting the peak area ratios of warfarin to quercetin against plasma concentrations. The lower limit of quantification (LLOQ) was determined as the concentration of the analyte with a signal-to-noise ratio of 10.

### Precision and accuracy

The intra-day precision and accuracy of the method were assessed by determining the concentrations of QC samples five times on a single day, and the inter-day precision and accuracy were estimated by determining the concentrations of QC samples over three consecutive days. The relative standard deviation (RSD) and relative error (RE) were used to express the precision and accuracy, respectively.

### Extraction recovery and matrix effects

The extraction recovery was evaluated by comparing the peak areas obtained from extracted spiked samples with those of the post-extraction spiked samples. The matrix effects were evaluated by comparing the peak areas of the post-extraction spiked QC samples with those of corresponding standard solutions. These procedures were repeated for five replicates at three QC concentration levels.

### Stability

The short-term stability was evaluated by determining QC samples at room temperature for 12 h. The auto-sampler stability was detected in auto-sampler after preparation for 12 h. The freeze-thaw stability was determined through three freeze-thaw cycles on consecutive days. The long-term stability was assessed by storing the QC samples at −40 °C for 30 days.

### *In vivo* pharmacokinetic study

To evaluate the effects of andrographolide on the pharmacokinetics of warfarin, the rats were divided into two groups of six animals each. The test group was pretreated with andrographolide by oral gavage at a dose of 30 mg/kg/day for 7 days before the administration of warfarin. Then warfarin was orally administered to the rats by oral gavage at a dose of 0.5 mg/kg. Blood samples (250 μL) were collected into heparinized tubes via the *oculi chorioideae* vein at 0.083, 0.167, 0.33, 0.5, 1, 2, 4, 6, 8, 10, 12, 24 and 36 h after the oral administration of warfarin. The blood samples were centrifuged at 3500 rpm for 5 min. The obtained plasma samples obtained were stored at −40 °C until analysis.

### Effects of andrographolide on the metabolic stability of warfarin in rat liver microsomes

The effects of andrographolide on the metabolic stability of warfarin were investigated using rat liver microsomes incubation systems. The assay conditions and reaction mixtures were similar to those reported previously (Jia et al. 2018; Wang et al. 2017). In brief, except for the NADPH-generating system, 30 μL of rat liver microsome (20 mg/mL), 12 μL of warfarin solution (100 μM, final concentration of 1 μM) and 1098 μL of PBS buffer (0.1 M, *pH* 7.4) were added to the centrifuge tubes on ice. The reaction mixture was incubated at 37 °C for 5 min and then the NADPH-generating system (45 μL) was added. The effects of andrographolide on the metabolic stability of warfarin were investigated by adding 12 μL of andrographolide (1.5 mM, final concentration of 15 μM) to rat liver microsomes and preincubating them for 30 min at 37 °C, followed by the addition of warfarin. Aliquots of 100 μL were collected from the reaction volumes at 0, 1, 3, 5, 15, 30, and 60 min after the addition of warfarin, and 200 μL of ice-cold acetonitrile containing the IS was added to terminate the reaction. The subsequent sample preparation method was the same as the plasma sample preparation method, and the concentration of warfarin was determined by LC-MS/MS.

The *in vitro *half-life (*t*_1/2_) was obtained using the equation: *t*_1/2_ = 0.693/k.

### Data analysis

The pharmacokinetic parameters, including the area under the plasma concentration-time curve (AUC), maximal plasma concentration (*C_max_*), time of the maximal plasma concentration (*T_max_*), and the mean residence time (MRT), were calculated using the DAS 3.0 pharmacokinetic software (Chinese Pharmacological Association, Anhui, China).

The differences between the mean values were analyzed for significance using a one-way analysis of variance (ANOVA). Values of *p <* 0.05 indicated statistical significance.

## Results and discussion

### Method validation

**Figure 1.**
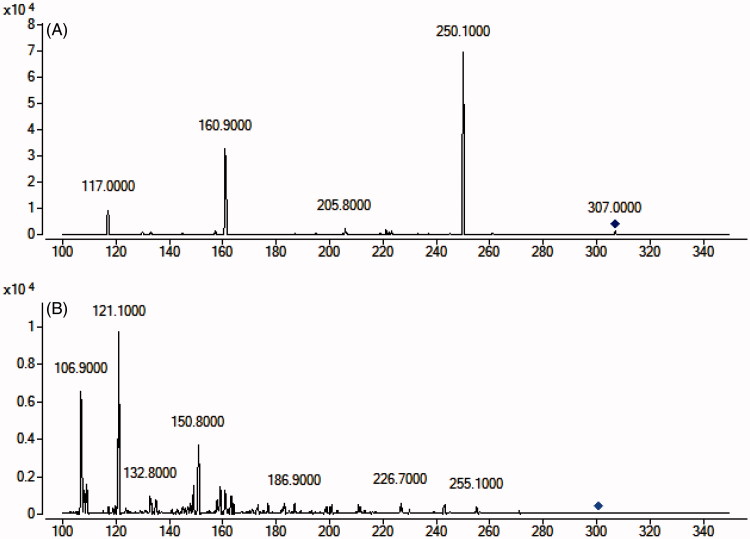
Mass spectra of warfarin (A) and quercetin (B).

**Figure 2.**
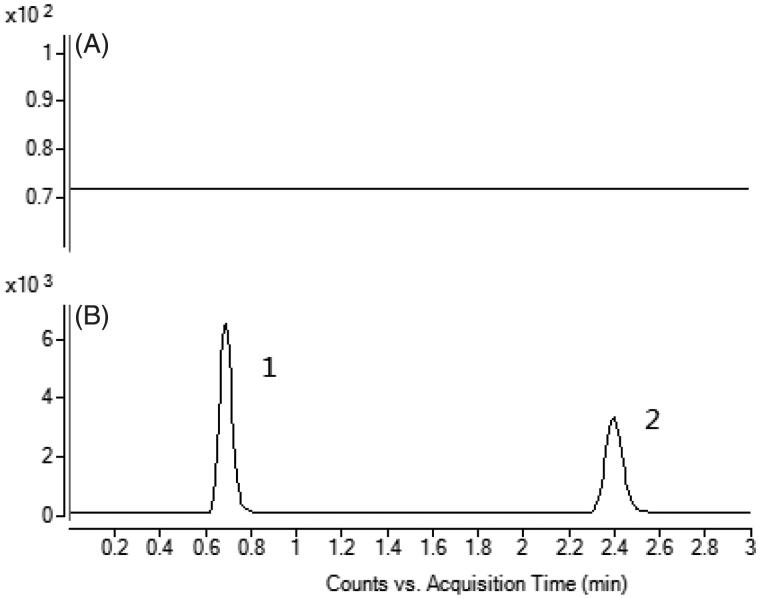
A: Representative chromatograms of blank plasma; B: Blank plasma spiked with warfarin and quercetin. (1) warfarin, (2) quercetin.

To develop a sensitive and accurate LC-MS/MS method for the determination of warfarin in rat plasma, quantitative analysis was performed using MRM mode owing to its high selectivity and sensitivity. The precursor and product ions were m/z 307.0 → 250.1 for warfarin, and 301.0 → 121.1 for quercetin. The mass ion spectra of warfarin and quercetin are shown in Figure 1. The MS/MS conditions were optimized to achieve better sensitivity and selectivity. To obtain the appropriate retention time and response, methanol, acetonitrile, water and formic acid were tested as mobile phases. After optimization, 0.1% formic acid was found to enhance the efficiency of ionization and obtain a better intensity than pure water for all compounds tested. Blank plasma, plasma spiked with warfarin and quercetin are shown in Figure 2. No significant interference was observed at the retention time of warfarin and quercetin in plasma samples.

The calibration curve for warfarin was constructed by plotting peak area ratios of the analyte to quercetin against plasma concentrations using a linear least-squares regression model. Linearity for determining warfarin in spiked rat plasma was prepared by nine calibration standards in five independent runs. The calibration curves were obtained with correlation coefficients (r) more than 0.999 between 10 and 5000 ng/mL of warfarin. The LLOQ was set at 10 ng/mL for warfarin in rat plasma samples.

The intra- and inter-day precision of the method was assessed at three concentration levels of spiked analyte in triplicate, and verified by determining the ratios of the peak areas of these compounds to the internal standard with relative standard deviation (RSD) as listed in Table 1. The precision of this method was not more than 10% relative standard deviations (RSD) for warfarin, indicating satisfactory precision and accuracy of the instrumentation.

**Table 1.** The intra-day and inter-day precision and accuracy of warfarin in plasma samples.

Spiked concentration (ng/ml)	Intra-day			Inter-day		
	Concentration measured (ng/ml)	Precision (%, RSD)	Accuracy (%, RE)	Concentration measured (ng/ml)	Precision (%, RSD)	Accuracy (%, RE)
20	18.68	7.36	−6.60	21.06	6.25	5.30
200	218.54	6.82	9.27	190.15	8.31	−4.93
4000	4220.89	5.94	5.52	3751.29	7.26	−6.22

To achieve high recovery efficiency in sample preparation, the direct precipitation method was used for its convenience and low matrix effects. Then, the extraction recovery of the precipitation solvents (methanol and acetonitrile) was investigated. The extraction efficiency of warfarin exceeded 90% by using acetonitrile as extraction solution, suggesting that it was an ideal precipitation agent. The matrix effects of warfarin were between 88.60% and 92.31%. These results indicate that the method was reliable and no obvious matrix effects were present.

As shown in Table 2, analyte stability was assessed under various conditions, and the results indicated that warfarin under these conditions were all stable in plasma samples (RE <10%).

**Table 2.** Stability of warfarin in plasma samples (*n* = 3).

Analyte	Plasma samples (ng/mL)	Stability (%, RE)				
		Short-term (12 h at room temperature)	Auto-sampler (12 h)	3 freeze-thaw cycles at −20 °C	Long-term (30 days at −20 °C)	
Warfarin	20	6.52	7.06	5.10	8.25	
	200	5.98	−6.85	7.32	−6.37	
	4000	−7.05	8.10	−5.50	9.31	

### Effects of andrographolide on the pharmacokinetics of warfarin

The pharmacokinetic parameters of warfarin were calculated using the noncompartmental method with the DAS 3.0 pharmacokinetic software (Chinese Pharmacological Association, Anhui, China). The pharmacokinetic parameters are shown in Table 3.

**Table 3.** Pharmacokinetic parameter of warfarin in rats after oral administration of warfarin (0.5 mg/kg; *n* = 6, Mean ± S.D.) with or without treatment of andrographolide (30 mg/kg/day for 7 days).

Parameter	Warfarin	Warfarin + Andrographolide
*T*_max_ (h)	2.02 ± 0.11	1.92 ± 0.15
*C*_max_ (μg/mL)	2.35 ± 0.25	3.32 ± 0.41*
*t*_1/2_ (h)	14.27 ± 2.67	22.73 ± 3.28*
AUC_(0-inf)_ (μg × h/mL)	60.58 ± 9.46	118.92 ± 18.08*
MRT (h)	13.49 ± 1.12	15.02 ± 1.28

*p < 0.05 indicate significant differences from the control.

**Figure 3.**
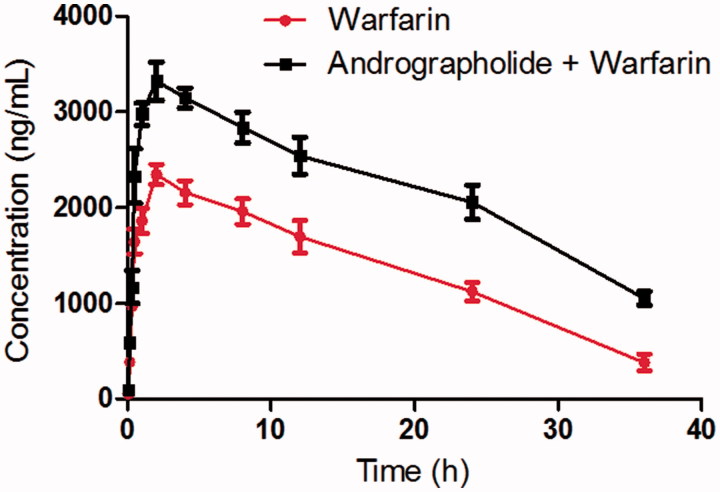
The pharmacokinetic profiles of warfarin in rats (six rats in each group) after the oral administration of 0.5 mg/kg warfarin with or without andrographolide pretreatment (30 mg/kg/day for 7 days). Each point represents the average ± S.D. of six determinations.

As shown in Figure 3, when the rats were pretreated with andrographolide, the *C*_max_ of warfarin increased from 2.35 ± 0.25 to 3.32 ± 0.41 μg/mL, and the difference was significant (*p* < 0.05). The AUC_(0-inf)_ warfarin was also significantly higher than that of the control (*p* < 0.05). The *t*_1/2_ of warfarin in rats pretreated with andrographolide was prolonged compared with the control (14.27 ± 2.67 *vs.* 22.73 ± 3.28 h), and the difference was also significant (*p* < 0.05).

These results indicated that andrographolide could increase the systemic exposure of warfarin in rats when andrographolide and warfarin were co-administered. Therefore, we think that the food-drug interaction between andrographolide and warfarin should be cautioned when they are co-administered. However, due to the pharmacokinetic differences between humans and rats, further *in vivo* system studies are needed to identify the interactions in humans.

Some other research has also found that when andrographolide was co-administered with other drugs such as naproxen, nabumetone and glyburide, their bioavailability would be increased (Balap et al. 2016, 2017; Samala and Veeresham 2016). Hovhannisyan et al (Hovhannisyan et al. 2006) have also investigated the effects of Kan Jang extract (a standardized fixed combination of extracts from *Andrographis paniculata* and *Eleutherococcus senticosus*) on the pharmacokinetics of warfarin in rats, and the results indicated that Kan Jang extract could increase the *C*_max_ of warfarin in rats, and however, the difference was not significant (*p* > 0.05). We infer that the concentration of andrographolide might be lower than that used in our study.

As andrographolide has been reported to inhibit the activity of CYP3A4 and CYP2C9 (Pekthong et al. 2008; Pan et al. 2011), warfarin was mainly metabolized by CYP3A4 and CYP2C9 in liver (Rougee et al. 2017; Zakharyants et al. 2017), and therefore, we speculated that andrographolide might increase the plasma concentration of warfarin by inhibiting CYP3A4 or CYP2C9-mediated metabolism.

### Effects of andrographolide on the metabolic stability of warfarin in rat liver microsomes

The effect of andrographolide on the metabolic stability of warfarin were investigated using rat liver microsomes. The metabolic half-life of warfarin was 23.5 ± 4.7 min, while the metabolic half-life was prolonged (38.7 ± 6.1 min) in the presence of andrographolide, and the difference was significant (*p* < 0.05). These results indicated that andrographolide could slow down the metabolism of warfarin in rat liver microsomes. As also shown Table 3, the t_1/2_ of warfarin in rats pretreated with andrographolide was prolonged compared with the control. These results suggested that andrographolide might decrease the metabolic clearance by inhibiting the activity of CYP3A4 and CYP2C9.

In summary, andrographolide could increase the systemic exposure of warfarin in rats when andrographolide and warfarin were co-administered. We infer that andrographolide might exert these effects mainly through slowing down the metabolism of warfarin in rat liver by inhibiting the activity of CYP3A4 and CYP2C9. Therefore, we think that the herb-drug interaction between andrographolide and warfarin might occur when they are co-administered.

## Conclusions

In conclusion, a sensitive and reliable LC-MS/MS method was developed and applied to determine the concentration of warfarin in rat plasma. The pharmacokinetic study indicated that andrographolide could significantly affect the pharmacokinetics of warfarin, and *in vitro,* rat liver microsomes metabolism experiments indicated that andrographolide might work by slowing down the metabolism of warfarin in rat liver. Therefore, the herb-drug interaction between andrographolide and warfarin should be cautioned when they are co-administered, and dose adjustment should also be considered.
